# Supplementary material for: Monitoring sick leave data for early detection of influenza outbreaks
Source: BMC Infect Dis. 2021 Jan 11;21:52. doi: 10.1186/s12879-020-05754-5 (PMC7799403; doi:10.1186/s12879-020-05754-5)
Supplement: Supplementary file 1 — Additional file 1. Figure S1: Incidence per 100,000 per week of influenza-like illness and sick leave in twelve French regions, 2010–2017. The Christmas and summer school holidays (increased worker leave periods) are shown at the bottom. Figure S2: Incidence of influenza-like illness and sick leave, 2015–2017, and alerts from the Sentinelles and the sick-leave models, in twelve French regions. The Christmas and summer school holidays (increased worker leave periods) are shown at the bottom. Table S1: Performance of the sick-leave model compared to the Sentinelles model. [file 12879_2020_5754_MOESM1_ESM.pdf]

Supplementary material

Figure S1: Incidence per 100,000 per week of influenza-like illness and sick leave in twelve French regions, 2010-2017. The Christmas and summer school holidays (increased worker leave periods) are shown at the bottom

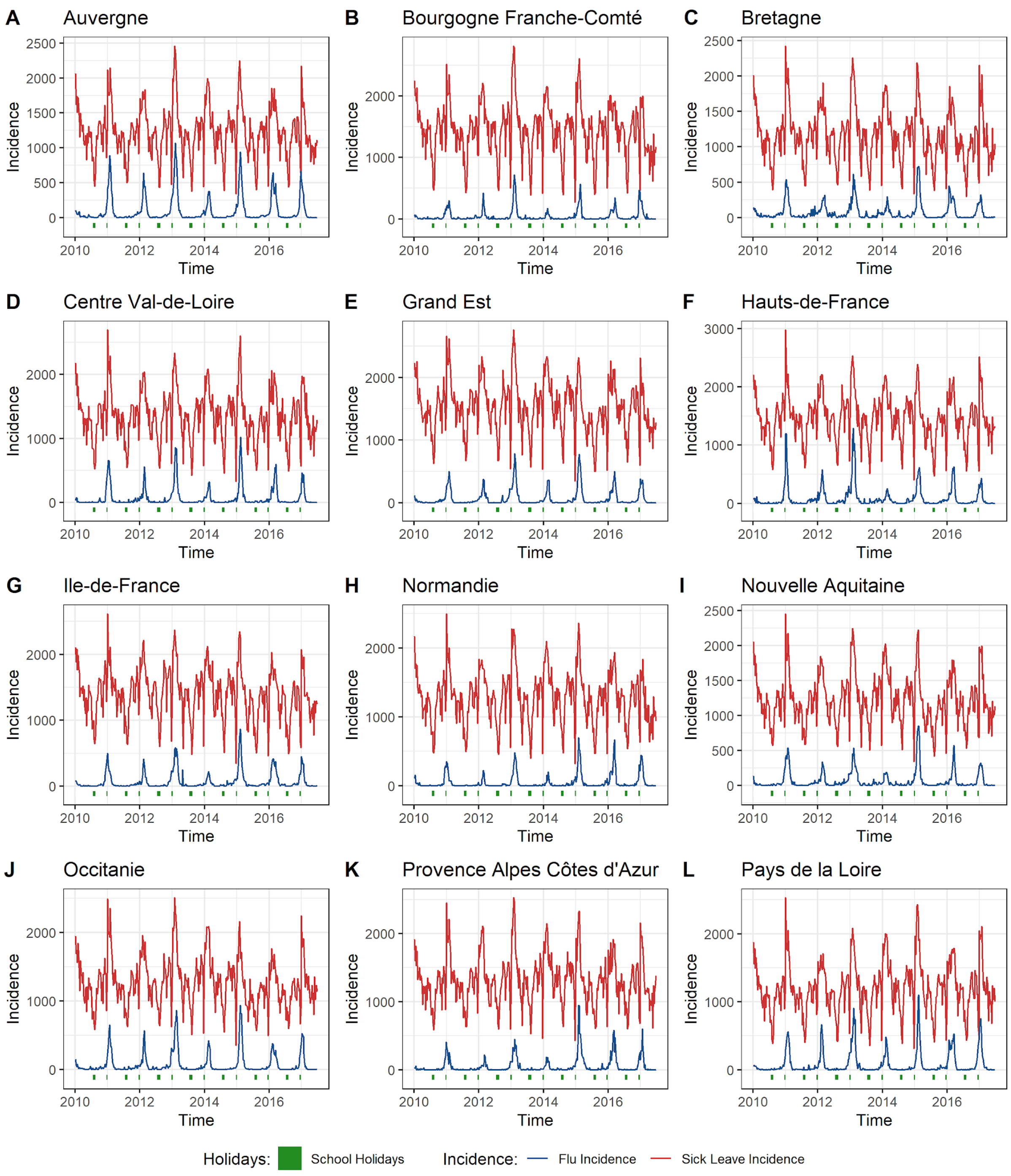

Figure S2: Incidence of influenza-like illness and sick leave, 2015-2017, and alerts from the Sentinelles *and the sick-leave models, in twelve French regions. The Christmas and summer school holidays (increased worker leave periods) are shown at the bottom*

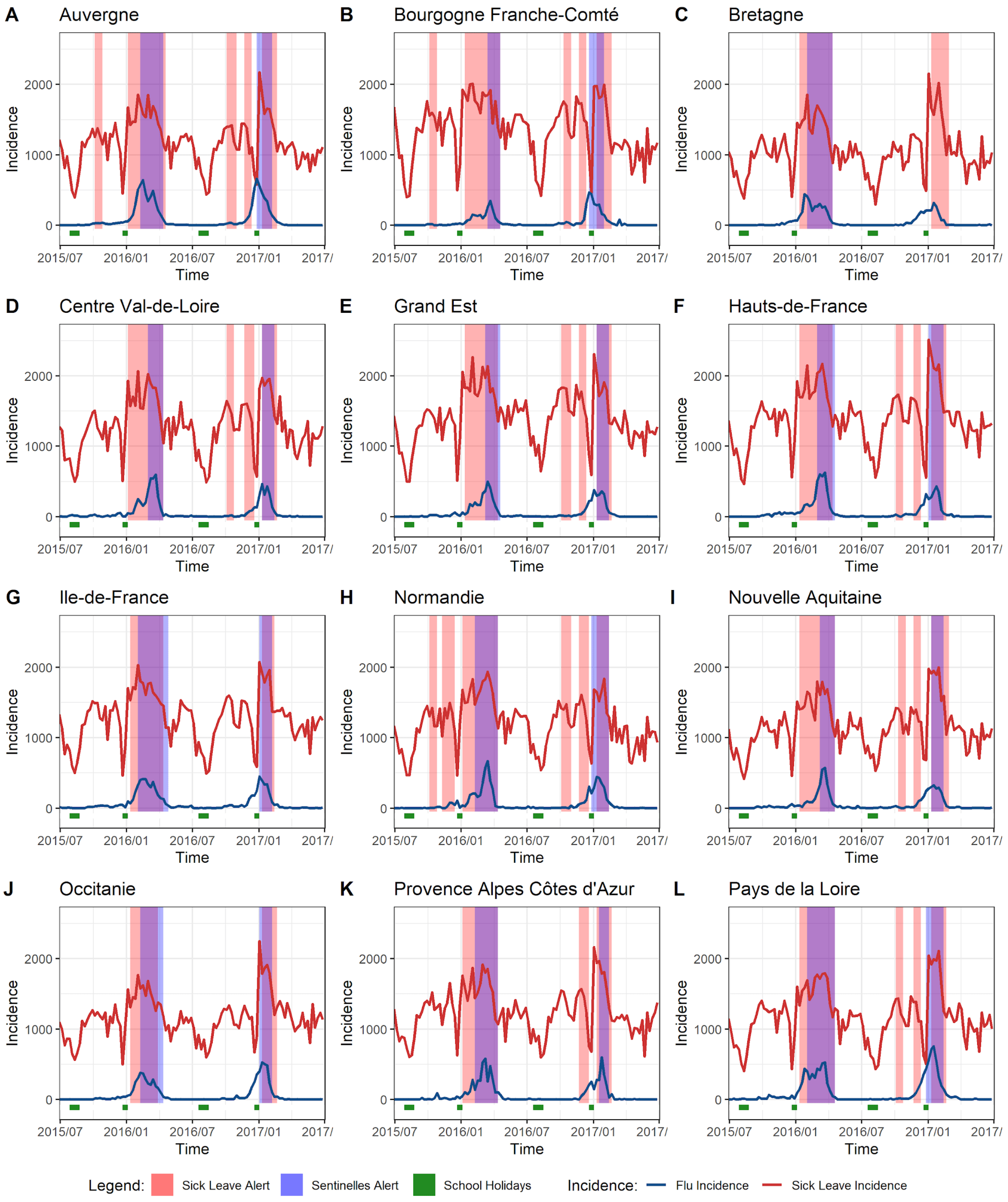

Table S1: Performance of the sick-leave model compared to the Sentinelles model

	Sensitivity	Specificity	Youden Index	Detection time (weeks) before ILI peak
Auvergne-Rhone-Alpes	0.87	0.81	0.68	1.5
Bourgogne-Franche-Comte	0.64	0.80	0.44	3
Bretagne	1	0.89	0.89	3
Centre-Val de Loire	1	0.83	0.83	4
Grand Est	0.91	0.84	0.75	4
Hauts-de-France	0.83	0.84	0.67	3
Ile-de-France	0.82	0.95	0.77	1
Normandie	0.93	0.80	0.73	1.5
Nouvelle-Aquitaine	1	0.85	0.85	4
Occitanie	0.79	0.95	0.74	1.5
Pays de la Loire	0.89	0.87	0.76	0.5
Provence-Alpes-Cote-d'Azur	1	0.87	0.87	3
Total mean	0.89	0.86	0.75	2.5

For each region, the values of these indicators are averaged over the two years evaluated.
